# Profile of patients with essential blepharospasm and hemifacial spasm in the two largest ophthalmology reference centers in Brazil

**DOI:** 10.5935/0004-2749.2022-0160

**Published:** 2023-03-20

**Authors:** Flávio A. Fowler, Cristina Yabumoto, Midori H. Osaki, Gustavo R. Gameiro, Janaina L. Brabo, Suzana Matayoshi, Regina C. R. S. Marinho, Tammy H. Osaki

**Affiliations:** 1 Department of Ophthalmology and Visual Sciences, Escola Paulista de Medicina, Universidade Federal de São Paulo, São Paulo, SP, Brazil; 2 Department of Ophthalmology, Universidade de São Paulo, São Paulo, SP, Brazil

**Keywords:** Blepharospasm, Hemifacial spasm, Meige syndrome, Blefarospasmo, Espasmo hemifacial, Síndrome de Meige

## Abstract

**Purpose:**

Information is scarce regarding the comprehensive profile of patients with essential blepharospasm and hemifacial spasm in Brazil. The present study aimed to assess the clinical features of patients with these conditions, followed up in two reference centers in Brazil.

**Methods:**

The study included patients with essential blepharospasm and hemifacial spasm, followed up at the Departments of Ophthalmology at *Universidade Federal de São Paulo* and *Universidade de São Paulo*. Apart from demographic and clinical features, past stressful events related to the first symptoms (triggering event), aggravating factors, sensory tricks, and other ameliorating factors for the eyelid spasms were assessed.

**Results:**

A total of 102 patients were included in this study. Most patients were female (67.7%). Essential blepharospasm was the most frequent movement disorder [51/102 patients (50%)], followed by hemifacial spasm (45%) and Meige’s syndrome (5%). In 63.5% of the patients, the onset of the disorder was associated with a past stressful event. Ameliorating factors were reported by 76.5% of patients; 47% of patients reported sensory tricks. In addition, 87% of the patients reported the presence of an aggravating factor for the spasms; stress (51%) was the most frequent.

**Conclusion:**

Our study provides information regarding the clinical features of patients treated in the two largest ophthalmology reference centers in Brazil.

## Abstract (pt)

**Objetivo:**

Há poucos dados sobre o perfil de pacientes com blefaroespasmo essencial e espasmo hemifacial no Brasil. O objetivo deste estudo é avaliar de forma mais abrangente as características clínicas dos pacientes portadores dessas doenças acompanhados em dois centros de referência em oftalmologia no Brasil.

**Métodos:**

Pacientes com blefaroespasmo essencial e espasmo hemifacial, acompanhados nos Departamentos de Oftalmologia da Universidade Federal de São Paulo e da Universidade de São Paulo foram incluídos neste estudo. Além dos dados clínicos e demográficos, foram avaliados também a presença de eventos estressantes relacionados ao início dos sintomas (evento desencadeante), fatores agravantes, truques sensoriais, e outros fatores de melhora.

**Resultados:**

Cento e dois pacientes foram incluídos no estudo. A maior parte dos pacientes era do sexo feminino (67,7%). Blefaroespasmo essencial foi a condição mais frequente, observada em 51 (50%) dos pacientes. Espasmo hemifacial correspondia a 45% dos casos, enquanto 5% dos pacientes apresentavam a Síndrome de Meige. 63,5% dos pacientes associaram o início dos sintomas com um evento estressante prévio. 76,5% dos pacientes relataram fatores de melhora para os espasmos; 47% relataram truques sensoriais. Além disso, 87% dos pacientes relataram fatores de piora; estresse (51%) foi o mais frequente.

**Conclusão:**

Este estudo fornece informações a respeito das características clínicas dos pacientes com blefaroespasmo essencial e espasmo hemifacial acompanhados nos dois maiores centros de referência em oftalmologia do Brasil.

## INTRODUCTION

Essential blepharospasm, the most frequent cranial dystonia, and hemifacial spasm are movement disorders affecting the facial muscles. These conditions usually develop and progress slowly over several years^(1,2,3,4)^.

Essential blepharospasm is associated with bilateral and involuntary eyelid spasms, resulting in intermittent periods of forced eyelid closure^(1,2,3)^. In many cases, there are precipitating factors, such as stress, bright light, emotion, and anxiety-provoking social situations^(1,5)^. Patients may adopt sensory “tricks”, purposeful maneuvers which, according to patients, temporarily reduce the spasms. Such “tricks” include local compression, singing, and deep breathing to reduce or mask the symptoms^(1,5)^. Blepharospasm associated with dystonic movements of other muscle groups in the face, neck, or limbs is known as Meige’s syndrome^(1)^.

Hemifacial spasm is not considered a form of dystonia but a peripheral movement disorder^(6)^. It is characterized by irregular involuntary tonic and/or clonic contractions of muscles innervated by the facial nerve^(1,2,4,7)^. Typically, unilateral facial muscle twitching initially affects the orbicularis oculi muscle. After months or years, the paranasal and perioral muscles become affected^(1,4)^. In contrast to essential blepharospasm, hemifacial spasms do not fade when the patient is asleep. Emotion and stress are also described as aggravating factors of the disease^(1,4)^.

Regarding epidemiologic features, essential blepharospasm has its peak onset in the sixth decade and occurs more frequently in women^(1,5)^. Women may also have a higher symptom frequency and severity^(8)^. Many environmental risk factors are thought to be involved in the onset of blepharospasm, such as urbanization, highly demanding jobs, and a stressful lifestyle^(9)^. Hemifacial spasm commonly has an insidious or subacute onset, usually earlier than essential blepharospasm, with a peak incidence in middle age and affects women more frequently than men^(1,4,7)^.

Although life expectancy seems unaffected in patients with essential blepharospasm and hemifacial spasm, the disease notably affects their quality of life^(8,10)^.

Large-series data are lacking regarding patients with essential blepharospasm and hemifacial spasm in Brazil. The present study aimed to assess the clinical profile of patients with these conditions followed up in Brazil’s two largest ophthalmology services. Apart from demographic and clinical features, past stressful events related to the first symptoms (triggering event), aggravating factors, sensory tricks, and other ameliorating factors for the eyelid spasms were assessed in the two centers.

## METHODS

This study complied with the ethical principles of the Declaration of Helsinki and was approved by the institutions’ review boards.

We included patients with essential blepharospasm, hemifacial spasm, and Meige’s syndrome, followed up at the Departments of Ophthalmology at *Universidade Federal de São Paulo* and *Universidade de São Paulo*.

The exclusion criteria included patients with less than 1-year follow-up, patients lost to follow-up, and those with secondary disorders.

The data examined were age, sex, diagnosis, age at onset of symptoms, comorbidities, and follow-up time. Additionally, participants underwent a structured interview in which they were asked about major stressful life events that had caused significant personal distress (such as the death of a loved one, divorce, or job loss), the presence of aggravating factors for the eyelid spasms, and the presence of sensory tricks or other ameliorating factors.

## RESULTS

The study included 102 patients with essential blepharospasm, hemifacial spasm, and Meige’s syndrome, followed up at the two centers.

**Figure 1:**
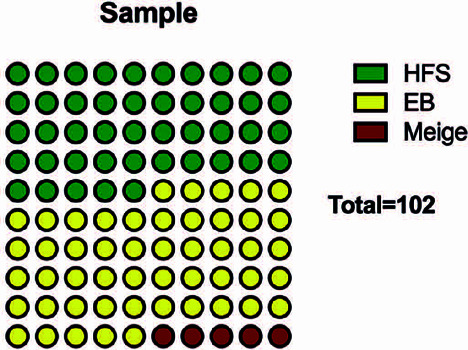
Distribution of the diseases in the two centers EB: essential blepharospasm, HFS: hemifacial spasm.

Essential blepharospasm was the most frequent disorder [51/102 (50%) patients]. Hemifacial spasm was found in 46 (45%) patients, while Meige’s syndrome composed a smaller part of the sample [5 (5%) patients] (Figure 1).

**Figure 2:**
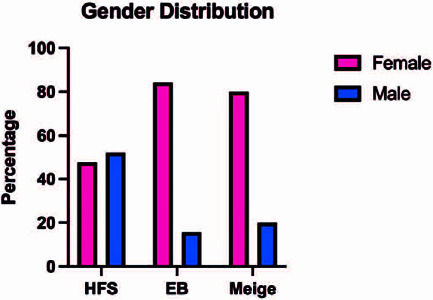
Sex distribution according to disease.

Concerning the sex of the patients, 33 were male (32.3%) and 69 were female (67.7%). Most patients with essential blepharospasm (84.3%) and Meige’s syndrome (80%) were female (Figure 2).

EB: essential blepharospasm, HFS: hemifacial spasm.

**Figure 3:**
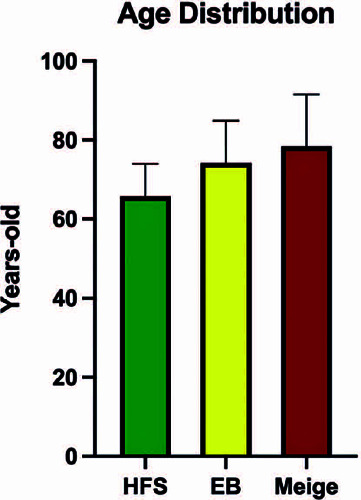
Mean age according to disease.

**Figure 4:**
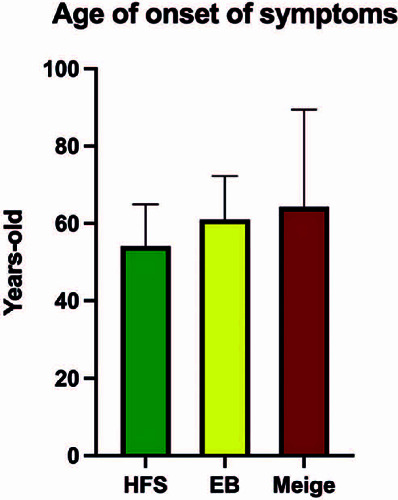
Age at onset of symptoms according to disease.

The patients’ ages ranged from 48 to 100 years, with a mean value of 70.63 ± 10.66 years. The mean age of patients with hemifacial spasm, blepharospasm, and Meige’s syndrome was 65.80, 74.22, and 78.40 years, respectively (Figure 3). The mean age at the onset of symptoms was 58.15 ± 12.18 years. The mean age at the onset of symptoms was 54.12, 61.00, and 64.25 years for hemifacial spasm, blepharospasm, and Meige’s syndrome, respectively (Figure 4).

EB: essential blepharospasm, HFS: hemifacial spasm.

EB: essential blepharospasm, HFS: hemifacial spasm.

The majority of patients (76.5%) had other comorbid chronic diseases; 50 patients (49.0%) had systemic hypertension, 16 (15.7%) had diabetes mellitus, 8 (7.8%) had a thyroid condition, 10 (9.8%) had high cholesterol, 4 (3.9%) had a heart disease, 7 (6.9%) had depression, 6 (5.9%) had glaucoma, and 17 (16.7%) had other comorbidities. Several patients had more than one comorbid disease.

The mean duration of disease was 12.30 ± 9.18 (3-40) years. The mean duration for each condition was 11.38 ± 9.76, 12.96 ± 8.33, and 14.75 ± 13.70 years for hemi facial spasm, essential blepharospasm, and Meige’s syndrome, respectively. The follow-up of the patients in the two centers varied between 14 months to 30 years, with a mean value of 7.53 ± 6.16 years.

**Figure 5:**
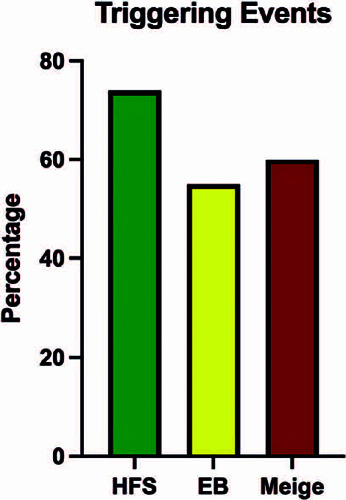
Presence of triggering events according to disease.

Regarding triggering events, 65 (63.5%) of the patients noted the onset of the disorder was associated with a past stressful event. Other reported events were prior diagnosis of another disease (21.6%), family disagreement (13.7%), death of a loved one (10%), and financial difficulty (7.8%). The presence of triggering events was reported by most patients with hemifacial spasm (74%), essential blepharospasm (55%), and Meige’s syndrome (60%) (Figure 5).

EB: essential blepharospasm, HFS: hemifacial spasm.

**Figure 6:**
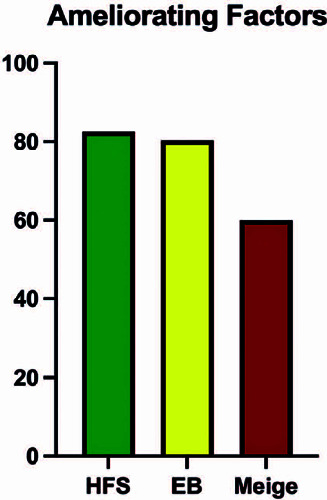
Presence of ameliorating factors according to disease

Ameliorating factors were reported by 76.5% of patients (80.4% for those with blepharospasm, 82.6% for those with hemifacial spasm, and 60% for those with Meige’s syndrome) (Figure 6). Rest (45.1%), local compression (34.3%), and cold water (9.8%) were the most frequently cited. Apart from local compression, additional 13 (12.8%) patients reported employing a sensory trick as an ameliorating factor, including singing (4.9%), concentrating on a pleasant activity (3.9%), speaking (2%), and whistling (2%).

EB: essential blepharospasm, HFS: hemifacial spasm.

Approximately 87% of the patients confirmed the presence of an aggravating factor. Stress was the most common [52 (51%) patients]; light and fatigue were reported by 26 (25.5%) and 8 (7.8%) patients, respectively.

## DISCUSSION

The present study analyzed the clinical features of esse ntial blepharospasm and hemifacial spasm in Brazil’s two largest ophthalmology services.

Essential blepharospasm was the most common disorder and was observed in 50% of our patients, corroborating with previous studies^(11)^. Women most frequently have cranial dystonias, especially in their fifties and sixties^(11,12,13,14,15)^. Our results agree with the literature, as we observed a female predominance (67.7%), and the mean age at symptom onset was earlier in hemifacial spasm than in essential blepharospasm (54.12 vs. 61.00 years)^(1,4,5,7)^.

Comorbid chronic diseases were similar to those found in a previous study, with a higher prevalence of hypertension, followed by diabetes and hypercholesterolemia^(15)^. Moreover, almost 6% of patients in this series had glaucoma (3% with essential blepharospasm and 3% with hemifacial spasm). Patients with essential blepharospasm and hemifacial spasm (on the affected side) have sustained abnormal eyelid tension from involuntary eyelid spasms^(16,17)^. Forced eyelid closure may lead to intraocular pressure peaks^(18)^. Glaucomatous optic neuropathy and corresponding visual field defects were observed in patients with essential blepharospasm. Furthermore, glaucoma-associated morphological findings were observed on the affected side in longlasting hemifacial spasm patients, suggesting that chronic repeated orbicularis contractions may be associated with glaucoma susceptibility^(18,19)^.

The prevalence of a major stressful triggering event preceding symptoms development was found in 63.5% of our patients. Prior studies reported rates varying from 24% to 72%^(5,12)^. Anderson et al.^(20)^ and Johnson et al.^(21)^ also reported the occurrence of a stressful event before the onset of symptoms. Johnson et al.^(21)^ observed that both types of facial spasms began within 1 year of a notably stressful life event in 70% of cases. Major life stressors and grief or depression might play a role in the pathogeneses of essential blepharospasm and hemifacial spasm in genetically susceptible patients^(21)^.

Sensory tricks are significant clinical features of essential blepharospasm^(5)^. Kilduff et al.^(22)^ and Loyola et al.^(6)^ observed that patients with hemifacial spasm also benefit from these alleviating maneuvers. Although little is known regarding the mechanism, sensory tricks act as relieving factors for the spasms^(6,22,23)^. Several sensory tricks have been described^(6,22)^. Local compression is one of the most frequently described tricks and was reported by 34.3% of our patients. Fantato et al.^(23)^, based on local compression as an alleviating maneuver for eyelid spasms, conducted a study that demonstrated an adjuvant effect of a simple spectacle-mounted device (*Pressop*) in those with essential blepharospasm. In our study, 47% of patients reported using a sensory trick. Kilduff et al.^(22)^ found that 52.7% of patients with essential blepharospasm and 44.6% with hemifacial spasm used ameliorating maneuvers in their series.

Almost 87% of the patients from this series confirmed the presence of an aggravating factor for the spasms. Stress was the most common, followed by light and fatigue. Anderson et al.^(20)^ reported that bright light might trigger or exacerbate symptoms in nearly 80% of patients with essential blepharospasm. In another study, patients with hemifacial spasm reported worse spasms in situations of fatigue and anxiety^(24)^.

The main limitation of this study is related to possible recall bias. Because these are chronic conditions, information regarding symptom durations might be inaccurate. In addition, analysis regarding treatment was not performed in the present study because of the different types of botulinum toxins used in the two centers. Moreover, the types of botulinum toxin received in each center varied periodically and precluded any meaningful comparisons.

In conclusion, the present study provides information regarding the clinical features of patients followed up at the two largest ophthalmology reference centers in Brazil. Future studies analyzing additional demographics of this population, such as stressful jobs, long working schedules, and information about rural vs. urban living, would be of value to better clarify the role of stressful routines as triggering/ aggravating factors in these patients.
